# Draft Genome Sequence of the Yeast Torulaspora quercuum Strain UCD657, Isolated from Soil in Ireland

**DOI:** 10.1128/mra.00952-22

**Published:** 2022-10-10

**Authors:** Blessing Igharo, Kevin P. Byrne, Heytham Ghnewa, Ryan P. Davey, Athaliah Fubara, Julia Welc, Shafik Oubihi, Luke Moore, Cian Smith, Eoin Ó Cinnéide, Sean A. Bergin, Conor Hession, Kenneth H. Wolfe, Geraldine Butler

**Affiliations:** a School of Biomolecular and Biomedical Science, Conway Institute, University College Dublin, Dublin, Ireland; b School of Medicine, Conway Institute, University College Dublin, Dublin, Ireland; Vanderbilt University

## Abstract

Torulaspora quercuum is an ascomycete yeast first isolated in 2009. Here, we present the genome sequence of T. quercuum isolate UCD657, which was isolated from soil in Ireland. This genome is 10.4 Mb and was assembled into 8 chromosome-sized scaffolds of >1 Mb in size, plus a mitochondrial genome scaffold.

## ANNOUNCEMENT

Three isolates of T. quercuum were first identified from the oral cavities of healthy volunteers in Tibet, with a fourth isolated from oak leaves in northern China (1). T. quercuum is related to Torulaspora delbrueckii, which has long been associated with winemaking (2). We identified T. quercuum isolate UCD657 from soil collected in Adamstown, Dublin, Ireland, near a broadleaf lime tree (GPS coordinates 53.337822, −6.458743). Soil material was passaged twice in 9 mL liquid yeast extract-peptone-dextrose (YPD) containing chloramphenicol (30 μg/mL) and ampicillin (100 μg/mL) and cultured on YPD plates at 30°C. The species was identified from single colonies by PCR amplification and Sanger sequencing of the internal transcribed spacer (ITS) region of its ribosomal DNA locus (accession number OP214351), which is 99% identical to that of T. quercuum strain CBS 11403 (AS 2.3768) (accession number FJ888524) (1).

For short-read sequencing, total genomic DNA was extracted from a YPD culture using phenol/chloroform (3) and dissolved in 150 μl water. Libraries were generated and sequenced by BGI Tech Solutions (Hong Kong). One microgram DNA was fragmented using Covaris, size selected (200 to 400 bp) using magnetic beads, end repaired, and 3′ adenylated, and primers were ligated. Fragments were amplified by PCR and heat denatured and circularized using the splint oligonucleotide sequence. The library was amplified with phi29 to make DNA nanoballs (DNBs). The DNBs were loaded on a patterned nanoarray, and 150 bases were sequenced from each end using a combinatorial probe-anchor synthesis (cPAS) on a DNBSeq-G400, yielding ~6.1 million read pairs. Default parameters were used for all methods. Adapters and low-quality reads were removed first by BGI using SOAPnuke (4) and subsequently using Skewer version 0.2.2 (5). For long-read sequencing, genomic DNA was prepared using a Genomic-tip 100/G kit (Qiagen). Libraries were generated using the rapid barcoding kit (product number SQK-RBK004) from Oxford Nanopore Technologies (ONT) and cleaned with AMPure XP magnetic beads. The flow cell priming kit (product number EXP-PLP002) was used to prime a fresh MinION R9.4.1 flow cell, and the libraries were sequenced using MinKNOW version 4.1.22 on a MinION Mk1C. Raw data were base called using Guppy version 4.2.2 +effbaf8 (using the fast model [dna_r9.4.1_450bps_fast.cfg]) (ONT) and demultiplexed using qcat version 1.1.0 (ONT) with default settings. NanoFilt (version 2.3.0) (6) was used to select reads (minimum quality of ≥7 and minimum length of ≥1,000 bp), which retained 227,483 reads with an *N*_50_ of 9,410 bp.

The genome was assembled from the long reads using Canu (version 2.2) (7), followed by five rounds of error correction with the DNBseq short reads using NextPolish (8). Five contigs of <51 kb (collapsed rDNA and telomeric regions) were removed, leaving 8 chromosome-sized contigs of >1 Mb in size and a circular mitochondrial genome (33,865 bp, manually edited, accession number OX291449.1). The total size of the genome was 10.4 Mb, the *N*_50_ value was 1.2 Mb, the *L*_50_ value was 4 contigs, and the G+C content was 41.3%. The largest contig was 1.9 Mb. Using BUSCO version 5.1.2, genome completeness was estimated at 98% (compared to the Ascomycota lineage data set).

### Data availability.

This whole-genome shotgun project has been deposited at DDBJ/ENA/GenBank (BioProject accession number PRJEB55421). The version described in this paper is version 1. The raw reads were deposited at SRA (accession numbers ERX9629580 and ERX9629581). The ITS sequence accession number is OP214351.

## References

[1] Wang QM, Xu J, Wang H, Li J, Bai FY. 2009. *Torulaspora quercuum* sp. nov. and *Candida pseudohumilis* sp. nov., novel yeasts from human and forest habitats. FEMS Yeast Res 9:1322–1326. doi:10.1111/j.1567-1364.2009.00567.x. 19751217

[2] Silva M, Pontes A, Franco-Duarte R, Soares P, Sampaio JP, Sousa MJ, Brito PH. 17 March 2022. A glimpse at an early stage of microbe domestication revealed in the variable genome of *Torulaspora delbrueckii*, an emergent industrial yeast. Mol Ecol. doi:10.1111/mec.16428. 35298044

[3] Dymond JS. 2013. Preparation of genomic DNA from *Saccharomyces cerevisiae*. Methods Enzymol 529:153–160. doi:10.1016/B978-0-12-418687-3.00012-4. 24011043

[4] Chen Y, Chen Y, Shi C, Huang Z, Zhang Y, Li S, Li Y, Ye J, Yu C, Li Z, Zhang X, Wang J, Yang H, Fang L, Chen Q. 2018. SOAPnuke: a MapReduce acceleration-supported software for integrated quality control and preprocessing of high-throughput sequencing data. Gigascience 7:1–6. doi:10.1093/gigascience/gix120. PMC5788068 29220494

[5] Jiang H, Lei R, Ding S-W, Zhu S. 2014. Skewer: a fast and accurate adapter trimmer for next-generation sequencing paired-end reads. BMC Bioinformatics 15:182. doi:10.1186/1471-2105-15-182. 24925680 PMC4074385

[6] De Coster W, D’Hert S, Schultz DT, Cruts M, Van Broeckhoven C. 2018. NanoPack: visualizing and processing long-read sequencing data. Bioinformatics 34:2666–2669. doi:10.1093/bioinformatics/bty149. 29547981 PMC6061794

[7] Koren S, Walenz BP, Berlin K, Miller JR, Bergman NH, Phillippy AM. 2017. Canu: scalable and accurate long-read assembly via adaptive k-mer weighting and repeat separation. Genome Res 27:722–736. doi:10.1101/gr.215087.116. 28298431 PMC5411767

[8] Chen Z, Erickson DL, Meng J. 2021. Polishing the Oxford Nanopore long-read assemblies of bacterial pathogens with Illumina short reads to improve genomic analyses. Genomics 113:1366–1377. doi:10.1016/j.ygeno.2021.03.018. 33716184

